# [^18^F] FDG uptake in patients with spondyloarthritis: correlation with serum inflammatory biomarker levels

**DOI:** 10.1186/s13550-023-00964-9

**Published:** 2023-02-15

**Authors:** Yuri Manabe, Takashi Norikane, Yuka Yamamoto, Mitsumasa Murao, Hiromi Shimada, Risa Wakiya, Shusaku Nakashima, Hiroaki Dobashi, Yoshihiro Nishiyama

**Affiliations:** 1 Department of Radiology, Faculty of Medicine, Kagawa University, 1750-1 Ikenobe, Miki-cho, Kita-gun, Kagawa, 761-0793 Japan; 2 Division of Hematology, Rheumatology and Respiratory Medicine, Department of Internal Medicine, Faculty of Medicine, Kagawa University, Kagawa, Japan

**Keywords:** FDG, PET, Spondyloarthritis, CRP, MMP-3

## Abstract

**Background:**

We aimed to evaluate the correlation between 2-deoxy-2-[^18^F]fluoro-D-glucose (FDG) uptake and disease activity assessed by serum inflammatory biomarker levels in patients with spondyloarthritis (SpA).

**Methods:**

A total of 36 SpA patients (24 untreated and 12 treated) were examined using FDG positron emission tomography (PET)/computed tomography and classified into axial SpA (axSpA) and peripheral SpA (pSpA). FDG uptake was evaluated in 23 regions of the body and scored as follows: 0 = less than liver uptake; 1 = more than or equal to liver uptake; and 2 = more than or equal to twice liver uptake. A score of 1 or 2 was considered positive. The number of positive regions and the total score were counted in each patient. The maximum standardized uptake value (SUVmax) was calculated for each region, and maximum SUVmax (MaxSUVmax) was used as a representative value. Correlation of PET findings with serum inflammatory biomarker levels, including C-reactive protein (CRP), erythrocyte sedimentation rate, and matrix metalloproteinase 3 (MMP-3), was analyzed.

**Results:**

All but two patients had at least one positive lesion. PET indices correlated significantly with most of the serum inflammatory biomarker levels in untreated SpA, but not in treated SpA. Further, MaxSUVmax, number of positive regions, and total score correlated significantly with CRP (all *P* values < 0.001), and the number of positive regions (*P* = 0.012) and total score (*P* = 0.007) correlated significantly with MMP-3 in untreated pSpA. PET indices did not correlate with any serum inflammatory biomarker level in untreated axSpA.

**Conclusion:**

FDG uptake in untreated pSpA correlated significantly with serum inflammatory biomarker levels.

## Background

Rheumatoid arthritis (RA) and spondyloarthritis (SpA)—sub-types of inflammatory arthritis—are often hard to differentiate in clinical practice. While patients with RA are often positive for rheumatoid factor (RF), those with SpA tend to be negative for the same. Consequently, SpA is routinely referred to as seronegative spondyloarthropathy. Further, while RA may present with primary synovitis, its main manifestations include entheses and secondary synovitis [1].

SpA has been classified into two groups based on its predominant distribution of inflammatory arthritis: axial SpA (axSpA) that mainly involves the spine or sacroiliac joints and peripheral SpA (pSpA). A degree of overlap between axSpA and pSpA, however, is a common occurrence [2]. The axSpA is further sub-divided into radiographic axSpA (r-axSpA), which equals to ankylosing spondylitis (AS), and non-radiographic axSpA (nr-axSpA), based on whether there are structural changes in the sacroiliac joints on the radiograph [3].

Magnetic resonance imaging (MRI) is more sensitive than the conventional imaging using radiography for the assessment of sacroiliitis. Radiography can only detect definite sacroiliitis such as bone erosion, sclerosis, and ankylosing, while MRI can also detect acute phase sacroiliitis such as bone marrow edema and fat deposition [4, 5]. MRI can be used not only for the assessment of sacroiliitis but also for that of spondylitis or other peripheral arthritis; however, it has difficulties in assessing whole body joints.

Positron emission tomography (PET) with 2-deoxy-2-[^18^F]fluoro-D-glucose (FDG) is a well-established tool for the diagnosis, staging, and evaluation of therapies for various malignancies. FDG accumulation is observed not only in tumor cells but also in inflammatory tissues, which allows its utilization for the evaluation of inflammatory diseases, including rheumatic diseases [2, 6]. Furthermore, FDG PET allows whole body examination in contrast with MRI. SpA involves whole body joints, although sacroiliac joints are preferred. MRI has mainly been used for the assessment of sacroiliitis or spondylitis but not for that of peripheral arthritis. Therefore, FDG PET may be more advantageous than MRI for assessment of SpA. In fact, previous studies using FDG PET on SpA demonstrated its utility in detecting lesions, and assessment of FDG uptake distribution was useful for differentiation from other rheumatic diseases [7–13]. Moreover, whole-body FDG PET scanning can detect other coexisting diseases. SpA associates with a variety of comorbidities; above all, accurate detection of cardiovascular disease is important for management of SpA patients [3, 14, 15].

Various well-established methods are available for assessment of disease activity in RA patients, including the Disease Activity Score (DAS) such as the DAS28, the Simplified Disease Activity Index (SDAI), and the Clinical Disease Activity Index (CDAI) [16]. FDG uptake correlates with disease activity as assessed by these methods with serum inflammatory biomarker levels and clinical examination of RA patients [17, 18]. Elzinga et al. reported that FDG changes due to treatment correlate with disease activity earlier than serum inflammatory biomarkers in RA patients [19]. Although certain other methods have been developed for SpA, their application is limited to specific SpA types. For instance, the Bath Ankylosing Spondylitis Disease Activity Index (BASDAI) and the Ankylosing Spondylitis Disease Activity Score (ASDAS) are useful for the evaluation of AS disease activity. Similarly, the Disease Activity Index for Psoriatic Arthritis (DAPSA), the Psoriatic Arthritis Joint Activity Index (PsAJAI), and the Composite Psoriatic Disease Activity Index (CPDAI) are employed for psoriatic arthritis (PsA), a type of pSpA [3, 15]. Moreover, in SpA patients, any association between disease activity and FDG uptake is currently unknown.

One of the treatment aims of SpA is the inhibition of disease progression. It is thus important to maintain low disease activity and to assess accurate disease activity. The present study aimed to evaluate correlation if any, between FDG uptake and disease activity as assessed by serum inflammatory biomarker levels in SpA patients.

## Materials and methods

### Patients

We conducted a retrospective analysis on a part of the data obtained in a prospective FDG PET/computed tomography (CT) study for diseases not covered by health insurance. The research protocol was approved by our institutional ethics review committee, and the requirement for obtaining informed consent was waived. Medical records of patients who underwent FDG PET/CT between December 2011 and October 2022 were reviewed. The inclusion criteria were as follows: patients with SpA and patients who had records of serum inflammatory biomarker levels, including C-reactive protein (CRP), erythrocyte sedimentation rate (ESR), and matrix metalloproteinase 3 (MMP-3), that were measured within 30 days of the FDG PET/CT scan. The exclusion criteria were as follows: patients who had liver disease or other infectious diseases and patients who had a history of other chronic inflammatory and autoimmune diseases. A total of 36 patients (20 males, 16 females; mean age, 53 years; age range 12–81 years) were thus enrolled in this study. Among these, 10 patients had axSpA [AS (*n* = 5) and nr-axSpA (*n* = 5)], and 26 patients had pSpA [PsA (*n* = 6), reactive arthritis (ReA) (*n* = 2), arthritis related to inflammatory bowel disease (IBD) (*n* = 3), and undifferentiated spondyloarthritis (uSpA) (*n* = 15)]. Patients with SpA were clinically diagnosed by the rheumatologists using the respective classification criteria. Patients with AS fulfilled the 1984 modified New York criteria, and patients with nr-axSpA and other SpA fulfilled the Assessment of the SpondyloArthrits International Society (ASAS) classification criteria for axial and peripheral SpA [20–22]. The median interval time between the FDG PET/CT scan and procurement of laboratory data was 7 days (range, 0–29 days).

Based on treatment contents at the time of FDG PET/CT, patients were divided into an untreated and a treated group; the untreated group (*n* = 24) included patients taking no medication or non-steroidal anti-inflammatory drugs (NSAIDs), and the treated group (*n* = 12) included patients taking glucocorticoid or disease-modifying anti-rheumatic drugs (DMARDs).

### PET/CT imaging

FDG was produced using an automated synthesis system with HM-18 cyclotron (QUPID; Sumitomo Heavy Industries Ltd, Tokyo, Japan).

All scans were performed using a Biograph mCT 64-slice PET/CT scanner (Siemens Medical Solutions USA Inc., Knoxville, TN, USA), with an axial field view of 21.6 cm. Patients were instructed to fast for at least 5 h prior to FDG PET/CT imaging, and peripheral blood glucose levels were confirmed to be in the normal range prior to initiation of the procedure. Whole-body PET emission scanning (2 min per bed position) was performed 90 min after intravenous FDG injection (5 MBq/kg), and co-registered with an unenhanced CT of the same region (Quality Reference mAs, 100 mAs [using CARE Dose4D]; reconstructed slice thickness, 5 mm). The PET data were acquired in a three-dimensional mode and reconstructed with a baseline ordered-subset expectation maximization (OSEM) algorithm, thus incorporating correction with the point-spread function and time-of-flight model (2 iterations, 21 subsets). A Gaussian filter of full width at half-maximum of 5 mm was used as a post-smoothing filter.

### PET/CT data analysis

PET images including maximum intensity projection images and PET/CT fused images were visually assessed by a nuclear medicine physician and a board-certified nuclear medicine physician independently in a blinded manner. Any differences in opinions were resolved by consensus. FDG uptake of the 23 following regions (sternoclavicular and sternocostal joints; bilateral shoulder joints including acromioclavicular joints; bilateral elbows; bilateral hands including wrists; spine including vertebral bodies, zygapophysial joints, atlantoaxial joint and costotransverse joints; bilateral sacroiliac joints; pubic symphysis; bilateral ischial tuberosities; bilateral anterior superior iliac spines; bilateral hips; bilateral greater and lesser trochanters; bilateral knees and bilateral feet) was visually scored. The scoring pattern was as follows: 0 = less than liver uptake; 1 = more than or equal to liver uptake; and 2 = more than or equal to twice liver uptake. Among these, a score of 1 or 2 was considered positive. The number of positive regions, ranging from 0 to 23, was enumerated for each patient. The total score of the 23 regions was subsequently obtained for each patient so that the total score for a single individual ranged from 0 to 46.

The regions of interest (ROI) were then placed in the highest FDG uptake area in each of the 23 regions by a nuclear medicine physician. The standardized uptake value (SUV) was calculated using the following formula: SUV = *c*_dc_/(*d*_i_/*w*), where *c*_dc_ is the decay-corrected tracer tissue concentration (Bq/g); *d*_i_, the injected dose (Bq); and *w*, the patient’s body weight (g). The maximum SUV (SUVmax) was calculated for each ROI. The maximum SUVmax (MaxSUVmax) of the 23 regions was then utilized as a representative value for patient-based assessment.

### Statistical analysis

All statistical analyses were performed using SPSS Statistics version 28.0.1.0 (IBM). All semiquantitative data were expressed as mean ± SD. Data were analyzed for statistical significance using the Mann–Whitney U test, Chi-squared test, Fisher’s exact test, and Spearman’s correlation coefficient. Statistical significance was set at *P* < 0.05.

## Results

**Fig. 1:**
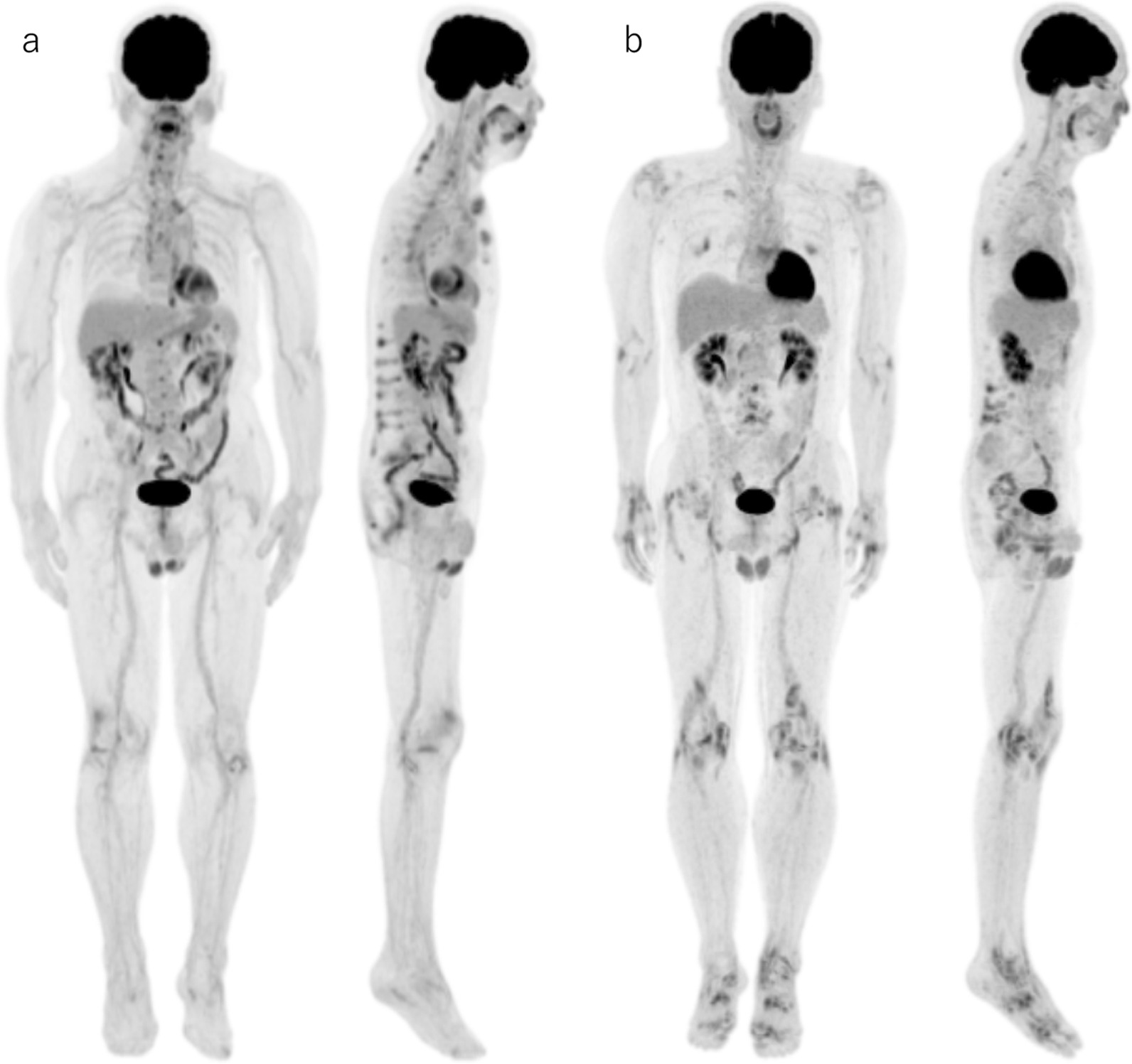
Typical FDG PET maximum intensity projection (MIP) images of patients with axial or peripheral spondyloarthritis. **a** Images of an 80-year-old man with axial spondyloarthritis (ankylosing spondylitis) showing increased uptake at the sternoclavicular and sternocostal, spinal, and sacroiliac joints. **b** Images of a 55-year-old man with peripheral spondyloarthritis (undifferentiated spondyloarthritis) showing increased uptake at each peripheral articular, including shoulders, elbows, wrists, hands, hips, knees, and feet and in the spine

**Fig. 2:**
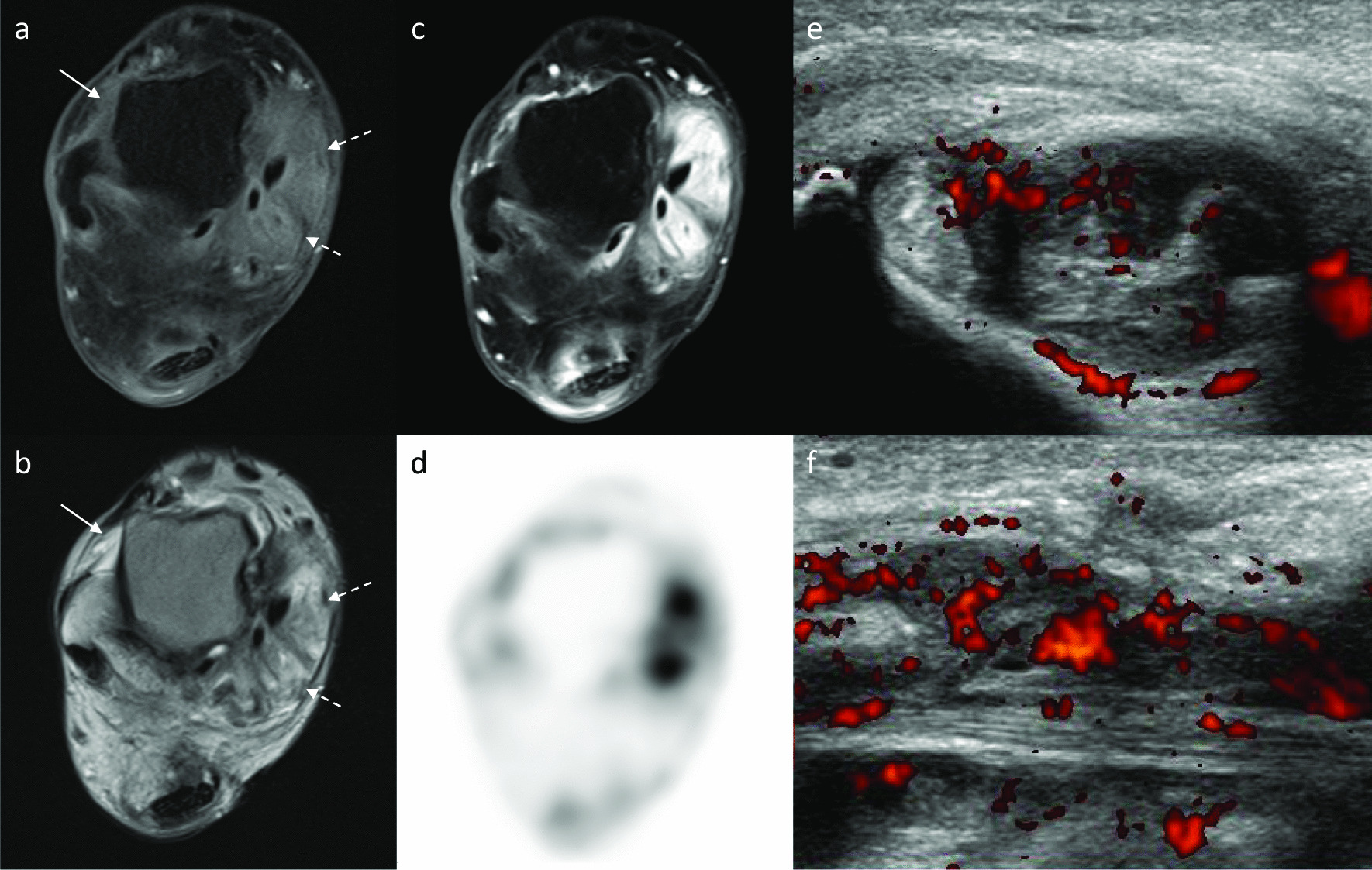
Images of right talocrural joint level in a 42-year-old woman with peripheral spondyloarthritis. **a** Axial T1-weighted and **b** axial T2-weighted images show synovial thickening anterior to talus (arrows) and around the medial flexor tendon (dashed arrows). These are enhanced in the fat-saturated contrast enhanced T1-weighted image (**c**), and FDG uptake is corresponding to synovial thickening (**d**). Color Doppler ultrasonographic images at the right medial flexor tendon (**e**; short axis, **f**; long axis) also show tenosynovitis as a hypoechoic area with heterogenous hyperemia

At least a single positive lesion was present in all but two patients. The other two included one patient with untreated axSpA and another patient with treated pSpA. The patients’ clinical characteristics, PET indices, and serum inflammatory biomarker levels are listed in Table 1. There were no significant differences in age, period from onset of SpA, sex, HLA-B27 positive rate, PET indices, and serum inflammatory biomarker levels between untreated and treated patients and between axSpA and pSpA in both untreated and treated patients. No significant difference was observed in the treatment period between axSpA and pSpA in treated patients. Typical axSpA and pSpA PET images are shown in Fig. 1. Clearly active synovitis has been identified on MRI and ultrasonography (US) at sites of FDG accumulation in pSpA patients (Fig. 2).

**Table 1:** Patients’ clinical characteristics, PET indices, and serum inflammatory biomarker levels

	Untreated (*n* = 24)		Treated (*n* = 12)	
	axSpA (*n* = 7)	pSpA (*n* = 17)	axSpA (*n* = 3)	pSpA (*n* = 9)
Age (year)	49 ± 22	49 ± 12	67 ± 17	58 ± 15
Period from onset of SpA (month)	45 ± 33	31 ± 38	56 ± 39	64 ± 65
Treatment period (month)	–	–	41 ± 34	53 ± 60
Sex				
Male	2	11	1	6
Female	5	6	2	3
HLA-B27 positive rate	14.3% (*n* = 7)	16.7% (*n* = 12)	0% (*n* = 1)	0% (*n* = 3)
PET indices				
MaxSUVmax	5.24 ± 5.81	5.63 ± 4.52	3.87 ± 1.42	5.21 ± 3.37
Number of positive regions	7 ± 5	11 ± 6	8 ± 6	12 ± 6
Total score	8 ± 6	14 ± 11	10 ± 8	15 ± 9
Serum biomarkers				
CRP (mg/dL)	0.70 ± 1.11 (*n* = 7)	1.99 ± 2.39 (*n* = 17)	0.76 ± 0.61 (*n* = 3)	0.65 ± 0.52 (*n* = 9)
ESR (mm/1 h)	27 ± 17 (*n* = 5)	34 ± 22 (*n* = 10)	37 ± 31 (*n* = 3)	25 ± 24 (*n* = 3)
MMP-3 (ng/mL)	70.6 ± 34.0 (*n* = 5)	120.6 ± 68.2 (*n* = 13)	94.3 ± 37.1 (*n* = 2)	90.7 ± 68.4 (*n* = 6)

Values are mean ± SD

*axSpA* Axial spondyloarthritis; *pSpA* Peripheral spondyloarthritis; *SUVmax* Maximum standardized uptake value; *CRP* C-reactive protein; *ESR* Erythrocyte sedimentation rate; *MMP-3* Matrix metalloproteinase 3

### Correlation between FDG uptake and serum inflammatory biomarker levels in untreated and treated SpA patients

**Fig. 3:**
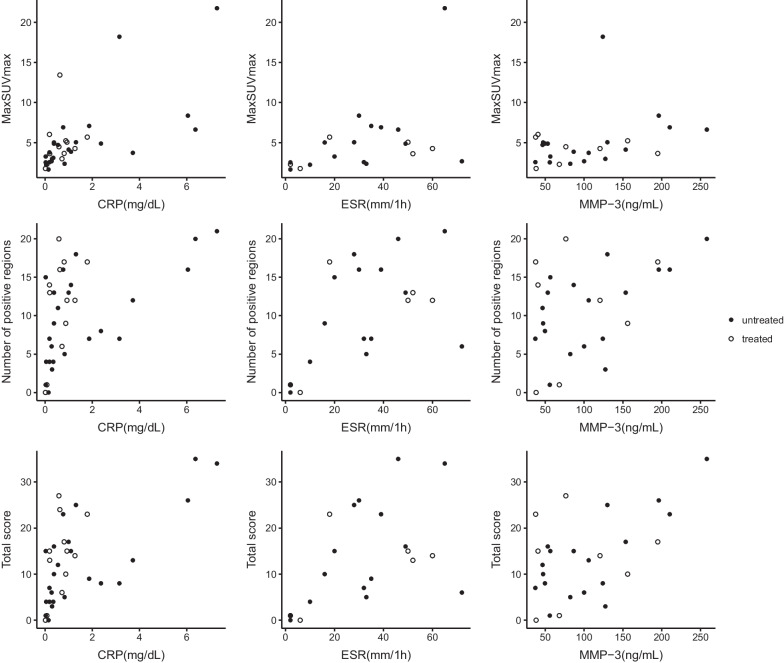
Correlations between PET indices and serum inflammatory biomarkers in untreated and treated spondyloarthritis patients

While PET indices correlated significantly with most serum inflammatory biomarker levels in the untreated SpA patients, they did not demonstrate correlation with serum inflammatory biomarker levels in treated SpA (Table 2, Fig. 3).

**Table 2:** Correlation between PET indices and serum inflammatory biomarker levels in untreated and treated SpA patients

	PET indices (*n* = 24)	CRP (*n* = 24)		ESR (*n* = 15)		MMP-3 (*n* = 18)	
		*ρ*	*P*	*ρ*	*P*	*ρ*	*P*
Untreated SpA	MaxSUVmax	0.771	< 0.001	0.527	0.043	0.449	0.062
	Number of positive regions	0.644	< 0.001	0.514	0.050	0.499	0.035
	Total score	0.684	< 0.001	0.534	0.040	0.551	0.018

	PET indices (*n* = 12)	CRP (*n* = 12)		ESR (*n* = 6)		MMP-3 (*n* = 8)	
		*ρ*	*P*	*ρ*	*P*	*ρ*	*P*
Treated SpA	MaxSUVmax	0.434	0.159	0.429	0.397	− 0.143	0.736
	Number of positive regions	0.326	0.301	0.493	0.321	0.156	0.713
	Total score	0.396	0.203	0.429	0.397	0.071	0.867

*SpA* Spondyloarthritis; *SUVmax* Maximum standardized uptake value; *CRP* C-reactive protein; *ESR* Erythrocyte sedimentation rate; *MMP-3* Matrix metalloproteinase 3

### Correlation between FDG uptake and serum inflammatory biomarker levels in untreated axSpA and untreated pSpA patients

**Fig. 4:**
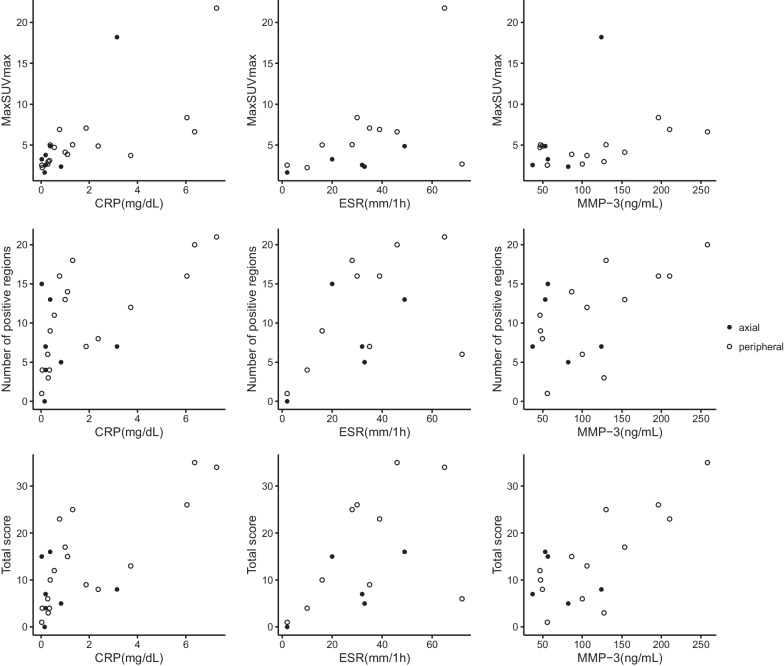
Correlations between PET indices and serum inflammatory biomarkers in axial and peripheral spondyloarthritis patients

PET indices did not correlate with levels of any serum inflammatory biomarker in untreated axSpA patients (Table 3, Fig. 4). In contrast, the MaxSUVmax, number of positive regions, and the total score correlated significantly with CRP (*P* values < 0.001) in untreated pSpA. Further, the number of positive regions (*P* = 0.012) and the total score (*P* = 0.007) correlated significantly with MMP-3. No significant correlations were observed between PET indices and ESR in these patients.

**Table 3:** Correlation between PET indices and serum inflammatory biomarker levels in untreated axSpA and pSpA patients

	PET indices (*n* = 7)	CRP (*n* = 7)		ESR (*n* = 5)		MMP-3 (*n* = 5)	
		*ρ*	*P*	*ρ*	*P*	*ρ*	*P*
Untreated axSpA	MaxSUVmax	0.468	0.289	0.600	0.285	0.300	0.624
	Number of positive regions	− 0.027	0.954	0.300	0.624	− 0.308	0.614
	Total score	0.180	0.699	0.600	0.285	− 0.200	0.747

	PET indices (*n* = 17)	CRP (*n* = 17)		ESR (*n* = 10)		MMP-3 (*n* = 13)	
		*ρ*	*P*	*ρ*	*P*	*ρ*	*P*
Untreated pSpA	MaxSUVmax	0.792	< 0.001	0.527	0.117	0.467	0.108
	Number of positive regions	0.798	< 0.001	0.529	0.116	0.669	0.012
	Total score	0.808	< 0.001	0.515	0.128	0.703	0.007

*axSpA* Axial spondyloarthritis; *pSpA* Peripheral spondyloarthritis; *SUVmax* Maximum standardized uptake value; *CRP* C-reactive protein; *ESR* Erythrocyte sedimentation rate; *MMP-3* Matrix metalloproteinase 3

## Discussion

SpA is a group characterized by chronic inflammatory arthritis, particularly sacroiliitis. Chronic inflammation causes progression of structural damages that are largely irreversible. These irreversible damages cause arthralgia, stiffness, and limited flexibility in SpA patients. These symptoms relate to the severity of SpA and can interfere with a patient’s quality of life (QOL). The aims of treatment are to alleviate symptoms, maintain and improve QOL, and decrease disease complications. It is difficult to achieve complete remission, and symptoms of relapse are often seen. Assessment of disease activity is essential for therapy because treatment may have to be switched or strengthened if disease activity is maintained at a high level or increases.

Assessment tools, including BASDAI or ASDAS, for determining disease activity in patients with SpA, particularly AS are based on the visual analogue scale and CRP or ESR value. Additional evaluation indices assess enthesitis, joint structure and function, and patient dysfunction in its entirety. Notably, these indices are only applicable to AS, and no suitable index is available for nr-axSpA, another type of axSpA. This has led to the utilization of the above mentioned indices for nr-axSpA, which has resulted in limited appropriate assessment of disease activity in SpA.

Although these assessment tools mainly comprise subjective patient-reported analogue scales, imaging modalities can objectively assess lesions. MRI of the spine and sacroiliac joints (SIJ) is used in clinical assessment, and various scoring systems using MRI for spine and SIJ have been developed. Zhang et al. demonstrated that MR DAS of SIJ, the Spondyloarthritis Research Consortium of Canada (SPARCC) MRI index, and apparent diffusion coefficient values in SIJ correlated significantly with the BASDAI score in AS patients [23]. However, another study reported that in axSpA patients there were no significant correlations between the SPARCC score and BASDAI and ASDAS, and no significant difference in the SPARCC score with high and low clinical DAS [24]. Furthermore, MR DAS is currently applied for axial skeletal lesions, but not for peripheral lesions. In peripheral lesions, US has been used for detecting enthesitis or synovitis in SpA, but to the best of our knowledge, there has been no study that has shown a relation between US and clinical DAS.

FDG PET can provide us whole body examination, and this is an advantage over MRI and US. Particularly, in pSpA patients with not only peripheral lesions but also axial lesions, it is advantageous that FDG PET can detect both axial and peripheral lesions in a single examination because the presence of axial lesions may affect treatment options. Furthermore, because FDG uptake has been observed in inflammatory tissues, FDG PET may be useful for early detection, similar to MRI. In fact, FDG PET has been used for the detection and evaluation of enthesitis or to distinguish it from similarly presenting rheumatic diseases, such as RA and polymyalgia rheumatica in SpA patients [7, 9, 11, 25]. Takata et al. demonstrated that SUVmax in lesions correlated with PsA DAS, including the Psoriasis Area and Severity Index and the DAS28 [26]. Abdelhafez et al. also reported that in PsA patients, FDG PET metrics showed a significant correlation with DAPSA [13]. These findings highlight the possible utility of FDG PET for the assessment of disease activity in PsA patients. In view of the fact that no reports are available on FDG PET in the context of disease activity in patients with other types of SpA, we evaluated the correlation between FDG uptake and disease activity assessed by serum inflammatory biomarker levels in SpA patients. Our findings demonstrated that FDG uptake in patients with untreated pSpA correlated with serum inflammatory biomarker levels.

While we conclusively demonstrated significant correlation between PET indices and serum inflammatory biomarker levels in untreated SpA patients, similar observations were not evident in treated SpA patients. Kaijasilta et al*.* showed that while sulfasalazine, a DMARD, reduces FDG PET-detectable inflammation in sacroiliac joints in axSpA, a concomitant decrease in inflammatory serum biomarker levels, such as CRP and ESR, did not occur [27]. This may add to the difficulty of establishing a relationship between FDG uptake and serum inflammatory biomarker levels during treatment. Moreover, in the present study, patients in the treated group were included based on their treatment contents at the time of PET/CT scanning only, and treatment periods and clinical effects were not considered. Therefore, the treated group included patients with different disease activities. Additional PET/CT scanning to assess treatment effect was not performed in the present study. Further studies are needed to validate whether FDG PET/CT can help assess treatment effect in SpA patients.

Significant correlation between CRP and all PET indices was evident in untreated pSpA but not in untreated axSpA. Similarly, MMP-3 expression correlated significantly with the number of positive regions and the total score in untreated pSpA, but not in untreated axSpA. Conventionally, SpA has been associated with entheses and RA with the synovium; however, recent reports suggest that both these conditions involve the synovium [28, 29]. Appel et al. also reported that synovitis is a hallmark of peripheral arthritis, and inflammation within the axial skeleton, chest wall, and hip is mostly associated with inflammation in the entheses or subchondral bone marrow [29]. Synoviocytes are potent sources of the cytokine interleukin 6 (IL-6), which is commonly induced by inflammation and known to stimulate production of acute-phase proteins, including CRP, serum amyloid A, and fibrinogen [30, 31]. Consequently, the predominant involvement of peripheral articular tissue in pSpA may result in high IL-6 secretion, thus reflecting synovitis. Further, predominant lesions of axSpA have little synovium. This may have affected the results that there were significant correlations between PET indices and CRP in untreated pSpA but not in untreated axSpA. The elevation of IL-6 secretion may be much higher in pSpA than that in axSpA, which may subsequently make the correlations between CRP and all PET indices more significant in untreated pSpA patients. Synovial inflammatory cytokines also induce expression of MMP-3, a proteinase, whose levels have been noted to correlate with disease activity in RA patients [32]. The correlation between MMP-3 and the number of positive regions and the total score in untreated pSpA may be reflective of synovitis in peripheral articular.

ESR, an inflammatory biomarker, often finds usage in the evaluation of disease activity scoring systems for RA, along with CRP. Beckers et al*.* reported correlation of ESR and CRP levels with FDG PET findings, including the number of FDG positive joints and the sum of SUVs in FDG positive joints in RA patients [33]. We did not observe any significant correlation between ESR and PET indices in untreated pSpA and axSpA patients. ESR levels are determined by various factors, and analysis is further complicated by the fact that pSpA patients often suffer from other diseases, including IBD, chlamydia infection, and psoriasis. Further studies on ESR are thus warranted, especially since we were unable to obtain sufficient ESR data.

Upon classification of SpA, all patients were classified as axSpA or pSpA based on ASAS classification criteria in the present study. However, the axial skeletal lesion preferentially occurring in axSpA may be involved in pSpA as well, and conversely, the peripheral lesion preferentially occurring in pSpA may also be involved in axSpA [2, 34]. The axial skeletal lesion in pSpA is often seen in PsA and arthritis related to IBD [35]. In the present study, there were also some patients with both axial and peripheral manifestations. Accordingly, if peripheral lesions included synovitis and our results were affected by synovitis, consideration of such a mixed type could alter the results.

This study had some further limitations, such as its retrospective design and its small sample size. These limitations may led to statistically non-significant correlations between any serum inflammatory biomarkers and PET indices in treated SpA and untreated axSpA, and between ESR and PET indices. While CRP data were available from all patients, ESR values were obtained from a limited number of patients. Additionally, visual PET indices may have underestimated axial skeletal lesions and disease activity in patients with axSpA, on account of the spine being considered one region. Finally, there was no follow-up on treatment effects or changes in disease activity after the PET/CT scan. In RA patients, FDG PET can predict treatment effects potentially earlier than serum biomarkers [19, 36]. We could not investigate treatment effects on SpA in FDG PET; however, its utility is expected. To overcome these limitations and validate our results, additional large-scale prospective studies are essential.

## Conclusion

The above-described preliminary findings reveal that while FDG uptake in untreated pSpA correlated significantly with serum inflammatory biomarker levels, the same was not true in untreated axSpA and treated SpA.

## Data Availability

The datasets used and analyzed during the current study are available from the corresponding author on reasonable request.

## Abbreviations

FDG: 2-deoxy-2-[^18^F]fluoro-D-glucose
SpA: Spondyloarthritis
PET: Positron emission tomography
axSpA: Axial SpA
pSpA: Peripheral SpA
SUVmax: Maximum standardized uptake value
MaxSUVmax: Maximum SUVmax
CRP: C-reactive protein
ESR: Erythrocyte sedimentation rate
MMP-3: Matrix metalloproteinase 3
RA: Rheumatoid arthritis
RF: Rheumatoid factor
r-axSpA: Radiographic axSpA
AS: Ankylosing spondylitis
nr-axSpA: Non-radiographic axSpA
MRI: Magnetic resonance imaging
DAS: The Disease Activity Score
SDAI: The Simplified Disease Activity Index
CDAI: The Clinical Disease Activity Index
BASDAI: The Bath Ankylosing Spondylitis Disease Activity Index
ASDAS: The Ankylosing Spondylitis Disease Activity Score
DAPSA: The Disease Activity Index for Psoriatic Arthritis
PsAJAI: The Psoriatic Arthritis Joint Activity Index
CPDAI: The Composite Psoriatic Disease Activity Index
PsA: Psoriatic arthritis
CT: Computed tomography
ReA: Reactive arthritis
IBD: Inflammatory bowel disease
uSpA: Undifferentiated spondyloarthritis
ASAS: The Assessment of the SpondyloArthritis International Society
NSAIDs: Non-steroidal anti-inflammatory drugs
DMARDs: Disease-modifying anti-rheumatic drugs
OSEM: Ordered subset expectation–maximization
ROI: Regions of interest
SUV: Standardized uptake value
US: Ultrasonography
QOL: Quality of life
SIJ: Sacroiliac joints
SPARCC: The Spondyloarthritis Research Consortium of Canada
IL-6: Interleukin 6
